# Experimental and theoretical studies of the efficiency of metal–organic frameworks (MOFs) in preventing aluminum corrosion in hydrochloric acid solution

**DOI:** 10.1186/s13065-024-01121-6

**Published:** 2024-01-28

**Authors:** Abd El-Aziz S. Fouda, Safaa Eldin H. Etaiw, Dina M. Abd El-Aziz, Ahmed A. El-Hossiany, Usama A. Elbaz

**Affiliations:** 1 Department of Chemistry, Faculty of Science, Mansoura University, Mansoura, 35516 Egypt; 2 Department of Chemistry, Faculty of Science, Tanta University, Tanta, Egypt; 3 Delta for Fertilizers and Chemical Industries, Talkha, Egypt

**Keywords:** Metal–organic frameworks (MOFs), Corrosion inhibition, Al, HCl, Langmuir isotherm

## Abstract

Aluminum corrosion inhibitors “{[CuI (CN)_2_(phen) CuII (CN)_2_(phen)]5H_2_O},(MOF1) and {[CuI(CN)_2_(phen)CuII(CN)_2_(phen)]5H_2_O}@TiO_2_ (MOF1@TiO_2_) were studied in one molar HCl solution”. The ML results for three different temperatures (25–45 °C) were compared with the results of PDP and EIS analyses. The adsorption of inhibitors on Al surfaces has been calculated and discussed by a Langmuir isotherm. The inhibitors that were created showed great effectiveness, with a noticeable increase in their inhibitory efficiency as the dosage was raised and the temperature was lowered. Inhibition efficiency each amounted to 88.6%, 84.5% at 400 ppm and 25 °C for MOF1@TiO_2_ and MOF1, respectively. Analyzing the polarization curves of synthesized inhibitors revealed that they were mixed-type inhibitors. Al was found to be surface inhibited when coated with a thin film of inhibitors, and “Al's surface morphology was assessed by different techniques such as scanning electron microscopy (SEM), energy dispersive X-ray (EDX) and atomic force microscope (AFM)”. “Theoretical models like quantum chemical and molecular dynamics simulation authenticated the experimental observation”. The MOFs exhibit exceptional corrosion resistance against Al when exposed to acidic environments, according to several tests.

## Introduction

Corrosion causes a lot of losses and damage to metals that have importance in life especially in the industry field due to the interaction of these metals electrochemically or chemically with the environment [1]. Many researches and practical experiments have been conducted over the past years to reduce corrosion by using some environmentally friendly corrosion inhibitors [2, 3]. One of the greatest ways to prevent the corrosion of metallic structures is to apply corrosion inhibitors, a technique that is frequently employed in global industries like the paint and coatings, water treatment, and oil and gas sectors. According to the medium treated, the type of metal utilized, and the type of corrosion experienced, a large amount of research and development has been done in producing corrosion inhibitors for different systems [4]. Corrosion inhibitors are utilized in a variety of industrial applications together with other additives including biocides and antiscalants. Al has many advantages in the field of industry because it is cheap, weight-low, has good heat/electrical conductance and is still considered one of the most widely used metals in engineering fields, after iron [5, 6]. The principle of Aluminum’s corrosion resistance in acidic and aqueous solutions is the formation of an oxide layer on its surface but in highly acidic or alkaline environments, this oxide film is subject to destruction [7]. It is known that in industrial cleaning operations, chemical and electrochemical analysis, and Al etching, it is therefore important and necessary to examine how hydrochloric acid inhibits Al corrosion in this acidic environment [8]. To reduce the surface corrosion of metals, corrosion inhibitors are used, which are divided into three main types: natural compounds, organic and inorganic compounds e.g., some coordination complexes, etc. [9, 10]. These inhibitors have many advantages, which are stability, low cost, and a strong effect on corrosion [11]. The researchers used nanotechnology to develop several corrosion inhibitors e.g., Titanium dioxide nan composites and silicon dioxide nan composites [12, 13]. Researchers have also found many ways to develop and increase the efficiency of inhibitors e.g., inhibitor concentration increasing, modification in composite structure, and synergistic effect [14]. The coordination complexes have a lot of advantages as corrosion inhibitors due to their supramolecular structure, heteroaromatic moieties, and richness in π-systems [15]. In the past three decades, researchers tended to study these nanosized composites due to their variety in organic linkers and their ability to build various structures with most of the elements in the periodic table [16]. Porous materials known as metal–organic frameworks (MOFs) are relatively new and are expanding quickly. The key feature of porous materials is their high degree of flexibility, which stems from the variety of ligands and metal nodes that can be added to them to give them new characteristics. This allows porous materials to be used for a wide range of applications, including drug delivery, water treatment, pollution, corrosion, and more. Many studies have been conducted on supramolecular substances and ideas in an effort to create new, more effective corrosion inhibitor systems. There are several recent papers on organic and polymeric corrosion inhibitors that make use of supramolecular ideas [17]. For example, a novel class of supramolecular Al terephthalate and its nanocomposite was used as an inhibitor against the AM60B magnesium alloy corrosion in an ethylene glycol solution containing chloride ions. The obtained results proved that the nanoparticles improved the inhibition efficiency from 86.52% to 90.8% [18]. It was demonstrated that cerium MOFs had a corrosion inhibition efficiency of 97% when used in CO_2_-rich brine solutions of API 5L X65 steel [19]. An effective and environmentally friendly inhibitor has been found in the supramolecular nanocomposite ZIF-8@ [Mo132]. During a study, it was proven that the compound contained inhibitory properties, making it an ideal candidate for preventing corrosion. The addition of 700 ppm of these components to a solution of hydrochloric resulted in an inhibition efficiency of 92.3% for Al. Various types of supramolecular compounds have been used as anticorrosion and data have been reported [20]. From the results obtained in the present work, we hope that it can be a useful addition to corrosion inhibitors with different advantages such as efficiency and novelty. And from the above, in this research, the supramolecular nanosized complex {[Cu_2_ (CN)_3_(phen)_3_]5H_2_O}, (SC)1 by incorporation of titanium dioxide nanoparticle, were synthesized. The prepared supramolecular nanosized composite was tested as anticorrosion inhibitor for Al in HCl solution. The synergistic effect between KI and new nanosized supramolecular was also tested for corrosion of Al in 1M HCl. MOFs are materials that combine inorganic and organic components through coordination bonds [21]. These materials have porous and crystalline surfaces, allowing them to be strongly adsorbent [22, 23]. Recent research has demonstrated that MOFs have effective corrosion inhibitor properties due to their heteroaromatic ligands [24]. Advantages and disadvantages of MOF compared to other micro and nanomaterials [25]:

### Disadvantages

One problem with pure MOFs is their low water stability, which results from water molecules attacking the coordination bonds that hold organic ligands and metal ions together. Pure MOF powders' insolubility, poor processability, and brittleness are other drawbacks that limit its usefulness in water treatment but there are several drawbacks of nanomaterials, including the issues of toxicology and the affordability of the manufacturing procedures.

### Advantages

MOFs are made up of organic linkers and metal-containing nodes that work together to provide adjustable pores, diversified functional sites, stable structures, and multi-functionality but Nanostructures create high crystallinity; offer a large surface area to increase the electrochemical reaction or molecule adsorption occurring at the solid–gas and/or solid–liquid interface.

In this study, MOF1 and MOF1@TiO_2_ were synthesized, characterized by XRD and examined as corrosion inhibitors because MOFs contain many active sites, a large specific surface area and changeable structure. Different methods, including ML procedure, polarization (PDP), and electrical impedance spectroscopy (EIS) tests were used to determine the effectiveness of these MOFs towards corrosion inhibition. An SEM, EDX and AFM analyses of the Al surface in HCl was also carried out to determine the formation film of the inhibitors on Al surface. A theoretical calculation of quantum chemical and Monte-Carlo simulations (MC) were computed and discussed.

## Experimental

### Chemicals

Each experiment was conducted with Al samples, before each test, the Al samples underwent a pre-treatment process involving the polishing of their surfaces using sandpaper with grit sizes of 200, 400, 600, 1200, and 2000 [26]. Rectangular Al samples measuring 2 × 2 × 0.1 cm and 1.0 × 1.0 × 0.1 cm were used for studying the mass loss and electrochemical properties of the Al, respectively. To create the corrosive control solution, commercial hydrochloric acid (HCl) with a concentration of 37% and density of 1.19 was mixed with distilled water. Inhibitor solutions were then prepared in concentrations ranging from 100 to 400 ppm.

#### Synthesis of {[CuI (CN)_2_(phen)CuII(CN)_2_(phen)]5H_2_O}(MOF1)

At room temperature, a solution of 99 mg (0.33 mmol) of K_3_[Cu(CN)_4_] in 30 ml H_2_O was added, under gentle stirring to a solution of 60 mg (0.334 mmol) of 1,10-phenanthroline (phen) in 20 mL acetonitrile. After 1-week green needle crystals resulted from the yellow solution, After filtration, subsequent washing with water and overnight drying, about 70 mg (51% referred to K_3_ [Cu (CN)_4_] of green crystals were obtained. Leff. = 1.95 BM, Anal. Calc. for 1 (C_39_H_34_N_9_O_5_Cu_2_): C, 56.0; H, 2.8; N, 15.0%. Found: C, 55.96; H, 2.7; N, 14.9% with chemical formula C_39_H_34_Cu_2_N_9_O_5_ and 835.06 g mol^−1^ molecular weight.

#### Synthesis of the nanocomposite {[CuI (CN)_2_(phen) CuII (CN)_2_(phen)] 5H_2_O}@TiO_2_ (MOF1@TiO_2_)

To produce a nanocomposite catalyst, a solution containing 1 g of nano-MOF1 and 35 mg of titanium dioxide in 20 ml of ethanol was subjected to ultrasonic radiation for 40 min at 60 watts, followed by 40 min of stirring in the dark to ensure effective absorption of the titanium dioxide by the MOF1. An exposed mixture of MOF1@TiO_2_ nanocomposite catalyst was filtered, washed several times in deionized water and ethanol, and then vacuum dried. An X-ray powder diffraction analysis and an infrared analysis were used to determine titanium loading on the MOF1 with chemical formula C_39_H_34_Cu_2_N_9_O_7_Ti and 432.9 g mol^−1^ molecular weight. Infrared (IR) analyses of MOFs were conducted using a Kappa CCd Enraf Nonius FR 90 four-circle goniometer at a temperature of 25 °C. The X-ray diffraction was performed using graphite monochromatic MoKa radiation [27].

### ML analysis

Standard techniques were used to measure ML [28]. Weighing of the abraded Al samples was completed three times using an analytical balance (accuracy of 0.1 mg) before exposure to an HCl environment containing 1.0 M HCl with and without various MOFs concentrations (100–400 ppm). In this case, the average value is the initial weight. In all acidic environments, samples were aerated and then removed, washed, dried, and weighed after 30 min of immersion. Three times are the means of weight measured after immersion tests. Experimental conditions included different temperatures ranging from 25 to 45 °C and a 3-h immersion time. Al rate of corrosion (${{\text{R}}}_{{\text{o}}}{\text{C}}$), was evaluated using Eq [29]:

1 $${\text{R}}_{{\text{o}}} {\text{C}} = { }\frac{{\Delta {\text{M}}}}{{{\text{A}}.{\text{t}}}}$$

Assuming $\Delta {\text{M}}$ is the ML of the Al specimens (mg), ${\text{A}}$ is the area of one sample (cm^2^), and ${\text{t}}$ is the immersion time (h). The ML (${\upeta }_{{\text{ML}}}\mathrm{\%}$) was calculated using Eq:

2 $${\upeta }_{{{\text{ML}}}} {\text{\% }} = { }\frac{{{\text{R}}_{{\text{o}}} {\text{C}}_{{\text{o}}} - {\text{ R}}_{{\text{o}}} {\text{C }}}}{{{\text{R}}_{{\text{o}}} {\text{C}}_{{\text{o}}} }}{\text{ x }}100$$

where ${{\text{R}}}_{{\text{o}}}{{\text{C}}}_{{\text{o}}}$ and ${{\text{R}}}_{{\text{o}}}{\text{C}}$ is the rate of corrosion (mg.cm^−2^.h^−1^) in the absence and presence of the inhibitor, respectively.

### Electrochemical techniques

Experiments were performed with different inhibitor doses (100–400 ppm) using a three-electrode cell design in 1.0 M HCl medium at ambient temperature. The working electrode is an Al electrode with a 1 cm2 exposed surface, and the reference electrode is an Ag/AgCl electrode that has been saturated with 3 M KCl. A platinum foil counter electrode with a surface area of about 1 cm2 is used [30]. Potentials are scanned at a rate of 0.2 mV/s over the voltage range of − 2.0–1.0 V. Through the charting of Tafel polarization curves with a potential range of ± 25 mV against the open circuit potential (OCP), corrosion current densities are determined in independent tests. Thirty minutes of immersion are required prior to doing any tests. Corrosion current densities were calculated by interpreting the Tafel's anodic and cathodic slopes. The formula used to compute the corrosion η_PDP_% is as follows [31]:

3 $${\upeta }_{{{\text{PDP}}}} {\text{\% }} = { }\frac{{{\text{i}}_{{{\text{corr}}}}^{0} - {\text{ i}}_{{{\text{corr}}}} }}{{{\text{i}}_{{{\text{corr}}}}^{0} }}{\text{ x }}100$$

In the absence and the presence of inhibitors the corrosion current densities (mA.cm^−2^) are denoted by i_corr_ and i^o^_corr_, respectively.

The electrochemical impedance spectroscopy (EIS) experiment was conducted in the 100 kHz-0.01 Hz frequency range using a system with amplitude of 0.010 V. A charge transfer measurement and the equation stated below were used to determine the ${\upeta }_{{\text{EIS}}}\mathrm{\%}$ [32]:

4 $${\upeta }_{{\text{EIS}}}\mathrm{\%}= \frac{{{\text{R}}}_{{\text{ct}}}- {{\text{R}}}_{{\text{ct}}}^{0}}{{{\text{R}}}_{{\text{ct}}}} \times \,100$$

The charge transfer resistance in the inclusion and exclusion of the inhibitor is shown by R_ct_0 and R^o^_ct_.

### Surface analysis

Analyzing Al surface is crucial to identify the morphology, proving the adsorption of MOFs and assessment of their impact as inhibitors. Our specimens were prepared by grounding the Al coupons to a grit of 4000 and then polished with a number of sand-papers. The prepared metal sheets were immersed in 1.0 M HCl solution for 24 h at 298 K without the addition of the inhibitors to evaluate the influence of corrosive medium on metal morphology. Analogous actions were conducted but with 400 ppm of inhibitor solutions. Comparison between the morphology of the samples attacked by the corrosive medium and those of the inhibited ones was done. These investigations were fulfilled by AFM (Model. FlexAFM3), SEM model A Jeol JSM-5400, and 15 kV as the acceleration voltage for EDX analysis. These analyses were performed in MAScIR-Rabat Foundation [33].

### Computational methods

#### Quantum chemical calculations

Using Material Studio (Software for modeling and simulating materials) version 7.0 semi-empirical approaches using the density functional theory (DFT), the entire quantum chemistry study has been conducted. The program is designed for quantum mechanics, molecular dynamics simulations, bioinformatics, chemo informatics, and computational chemistry. Advanced study on a variety of materials, including polymers and carbons, is conducted using this program. Semi-empirical methodology was used to calculate molecular orbitals. The molecules were optimized by choosing B3LYB (Becke-3-parameters-lee-yang-parr) with DNP functions while setting the fine quality. Fine convergence and global orbital cutoffs were utilized as well as setting water as solvent which impact the treatment via COSMO controls.

#### Monte-Carlo simulations (MC)

Using MC, the optimal positioning of MOFs inhibitors on the apparent of Al (111) was evaluated. According to the literature [34], it is believed that the Al (1 1 1) crystal surface is used in this simulation due to its most stable. The estimation module was initially used to carry out the geometrical optimization of water and the inhibitor molecule. Compass stimulation along with force field were implemented to MOFs on Al (1 1 1) optimized surface. The substrate-adsorbate system configuration space was searched using the Monte-Carlo approach to identify low-energy adsorption sites where the temperature gradually decreases.

## Results and discussion

### Characterization of MOF1 and MOF1@TiO2

#### A study of the properties of MOF1 and MOF1@TiO2 composites {[Cu^I^(CN)(phen)_2_.Cu^II^(CN)_2_.(phen)]0.5H_2_O}@TiO_2_

**Fig. 1:**
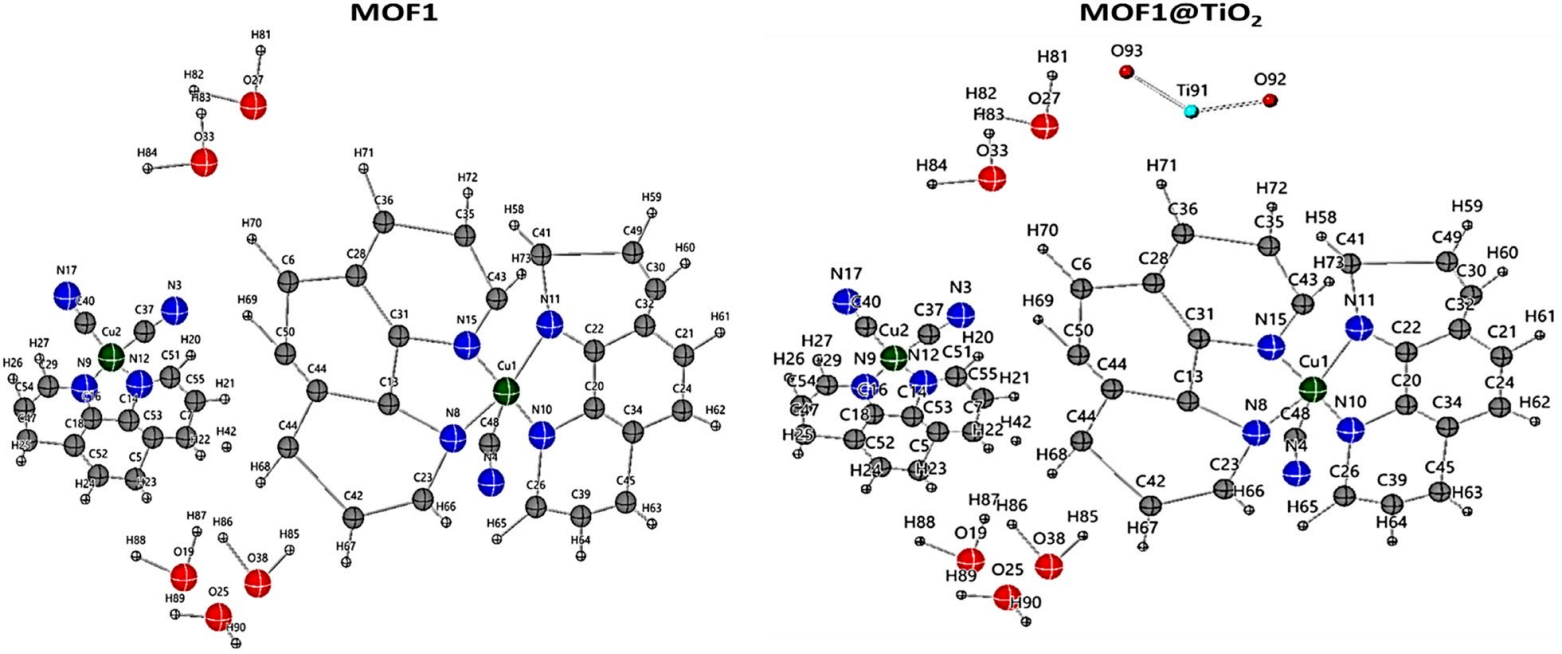
Asymmetric unit of Crystal data for the MOF1 and MOF1@TiO_2_

It has previously been demonstrated that individual crystals of the supramolecular system [[Cu_2_(CN)_3_(phen)_3_]5H_2_O] exhibit X-ray diffraction, which provides an opportunity to study the topology of supramolecular systems and the role of flexibility and ligand. This structure contains anionic mixed valence [Cu^I^ (CN)_2_(phen)]^−^ and [Cu^II^ (CN)(phen)_2_]^+^ moieties, with Cu^I^ adopting a distorted trigonal bipyramid and Cu^II^ exhibiting slightly distorted tetrahedral topology in addition to five water molecules which are connected with each other by hydrogen bonds, Fig. 1. The distortion of trigonal bipyramid observed for Cu^I^ topology is due to the hydrogen bonds between the cyanide group and the water molecules, N4–H27A = 3.10 A°, N4–H38B = 3.01 A°. On the other hand, for strictly square-planar complexes with D4h symmetry, the tetrahedrality is 0. For tetrahedral complexes with D2d symmetry, the tetrahedrality equals 90. In MOF**1** the angle of the two planes; C17Cu2C40 and N12Cu2N9, is 85.21 supporting the tetrahedral topology of Cu^II^. The water molecules create 1D-tape which consists of fused two four- and one six-oxygen member rings forming water cluster. In addition the water molecules are connected via hydrogen bonds forming 1D-chain [35]. The water molecules play a crucial role in forming the 3D structure via hydrogen bonding. The new nanoscale MOF1 is stable under air and light and was obtained as the only green nanosized crystalline product via ultrasound irradiation. It is soluble in DMSO, DMF, and ethanol, sparingly soluble in most organic solvents, and insoluble in water. Elemental analysis and single-crystal X-ray diffraction data support the chemical formula (C_39_H_34_N_9_O_5_Cu_2_) for MOF1. The MOF1@TiO2 nanocomposite was found to contain 11.43% titanium, as determined by the inclusion of Nano-titanium in the MOF1 structure.

#### IR analysis

**Fig. 2:**
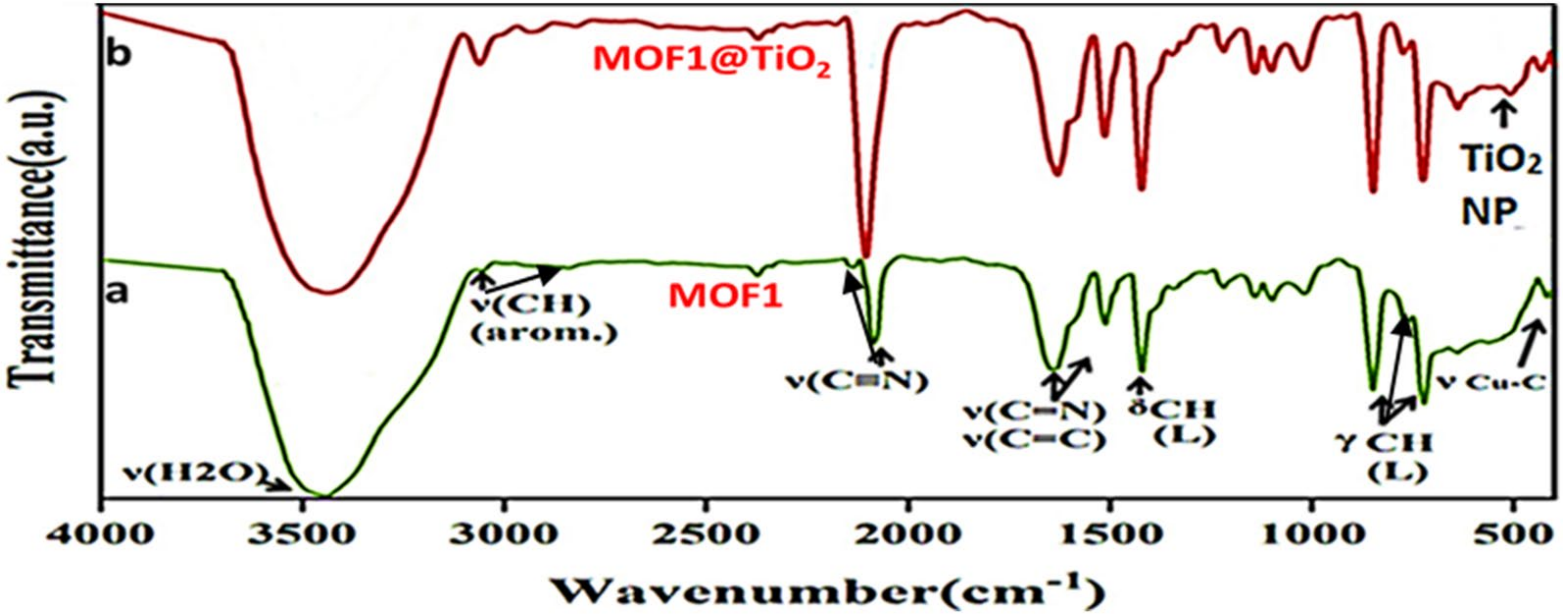
IR spectrums of MOF1 and MOF1@TiO_2_

Figure 2 represent the IR spectrum of MOF1 shows characteristic bands which corresponding to H_2_O, phen and CuCN building blocks at specific wavenumbers, such as 3444 cm^−1^ for ν(H2O), 3051 and 2842 cm^−1^ for ν(CH) arom, 2132 and 2088 cm^−1^ for ν(CN), 1646 and 1510 cm^−1^ for ν(C C) and (C N), 1420 and 1360 cm^−1^ for δCH phen, and 762, 722, and 638 cm^−1^ for γCH phen, as well as a band at 415 cm^−1^ for νCu C. These same bands were also observed in the IR spectrum of the MOF1@TiO_2_ nanocomposite, along with a band at 509 cm^−1^ that can be attributed to the presence of nano-titanium. This supports the conclusion that the nanoparticles of titanium are included in the cavities of MOF1.

#### XRPD analysis

**Fig. 3:**
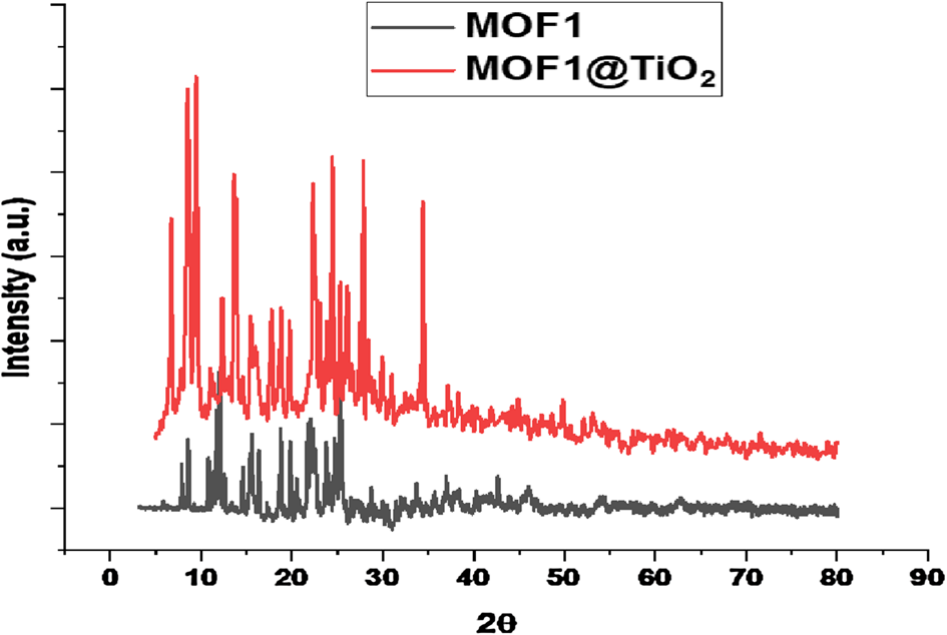
X-ray diffraction patterns of MOF1 and MOF1@TiO_2_

A peak that corresponds to Nano-titanium (2ϴ = 27) can be detected in the XRPD of MOF1@TiO_2_ nanocomposite, indicating it is well incorporated into MOF1 as shown in Fig. 3.

#### ML analysis

**Fig. 4:**
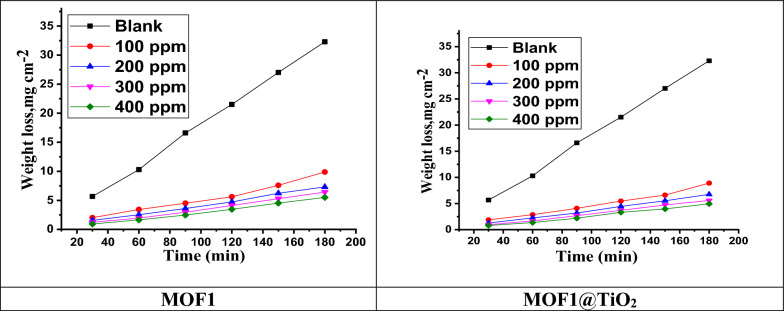
Plots of mass loss against time for Al in 1.0 M HCl with and without free acid, as well as with and without different MOF1 and MOF1@TiO2 dosages at 25 °C

In Fig. 4, MOFs protect Al against corrosion by reducing ML and enhancing inhibition efficiency (${\upeta }_{{\text{ML}}}\mathrm{\%}$). An ML test performed at 25 ^°^C demonstrated that as MOFs concentrations were increased, the ${{\text{R}}}_{{\text{o}}}{\text{C}}$ decreased, so the inhibition improved as more molecules were adsorbing onto the Al, thus decreasing HCl interactions. 400 ppm MOF1@TiO_2_ exhibited the highest inhibition efficiency (85.8%), while MOF1 demonstrated the highest (83.0%). Nitrogen, oxygen, and sulfur atoms donate electrons, as do double bonds, which improve the inhibitor's ability to shift electron pairs to (from inhibitor molecules) the unoccupied p-orbitals of Al atoms, controlling corrosion and/or preventing corrosion progression. Inhibitors become less effective at lower temperatures [36]. A temperature change from 25 to 45 ^°^C results in a decline in MOFs inhibition efficiency as shown in Tables 2 and 3. It appears that MOFs molecules remove from Al surfaces as temperatures rise, allowing Al to become unprotected by MOFs molecules, thus reducing corrosion (${\upeta }_{{\text{ML}}}\mathrm{\%}$).

#### Kinetic parameters of activation

Three hours at different MOF concentrations were used to study the impact of temperature (25–45 ^°^C). “Tables 1 and 2 compile information collected by ML measurement together with the corresponding % η. The corrosion rate of Al increases as temperature rises in acidic environments. It is common knowledge that when temperature rises, corrosion rate also rises. Corrosion inhibition rises with inhibitor concentration at each temperature. According to the data, the inhibitor works by adsorbing on the surface of Al and blocking the active sites to create a screen that protects the surface from acidic solutions. The surface becomes less protected as the temperature rises, and the inhibitor gradually loses its effectiveness as a result. We see that desorption rate exhibits parallel behavior to that of adsorption. It is possible to learn more about how the Al/HCl interface behaves by examining the effect of temperature on the kinetic process of corrosion in free acid and the presence of an inhibitor”. According to the relationship between corrosion rate (CR) calculated from ML measurements and 1/T, the Arrhenius rule can be represented as a straight line of the logarithm of the corrosion rate:

5 $${\text{CR}}\, = \,{\text{k exp}}{-}{\text{E}}_{a}^{*} /{\text{ RT}}$$

**Table 1:** An investigation of Al corrosion inhibitors activity at different temperatures in the presence and absence of MOF1

Conc., ppm	Temp.,^o^C	CR, mg/(cm^2^ min)	Θ*	η%
Blank	25	14.1 ± 0.4234	–	–
	35	24.5 ± 0.8232	–	–
	45	47.8 ± 0.9921	–	–
100	25	2.4 ± 0.001201	0.831	83.1
	35	5.1 ± 0.000881	0.796	79.6
	45	10.7 ± 0.002027	0.776	77.6
200	25	2.36 ± 0.001463	0.839	83.0
	35	4.9 ± 0.001154	0.800	80.0
	45	10.7 ± 0.001763	0.783	78.3
300	25	2.3 ± 0.001763	0.840	84.0
	35	5.2 ± 0.001452	0.803	80.3
	45	9.8 ± 0.001201	0.794	79.4
400	25	2.4 ± 0.00120	0.855	83.0
	35	5.1 ± 0.002081	0.806	80.6
	45	9.90.003511	0.794	79.4

Where Θ^*^ is the degree of surface coverage

**Table 2:** A study examining Al corrosion inhibitors in the absence and presence of MOF1@TiO2 at 400 ppm and their effectiveness at different temperatures

Conc., ppm	Temp.,^o^C	CR, mg/(cm^2^ min)	θ	η%
100	25	2.8 ± 0.001452	0.801	80.1
	35	5.0 ± 0.001452	0.796	79.6
	45	10.4 ± 0.08819	0.782	78.2
200	25	2.7 ± 0.001732	0.809	80.9
	35	5.0 ± 0.001201	0.796	79.6
	45	10.1 ± 0.00120	0.786	78.6
300	25	2.4 ± 0.00173	0.830	83.0
	35	4.2 ± 0.002603	0.824	82.4
	45	9 ± 0.010012	0.791	79.1
400	25	2.0 ± 0.00176	0.884	85.8
	35	4.2 ± 0.00173	0.829	82.9
	45	9.0.± 0.00120	0.812	81.2

where “k is the Arrhenius pre-exponential factor, R is the gas constant, E^*^_a_ is the apparent activation energy of the corrosion reaction, and T is the absolute temperature. CR is the corrosion rate, which is derived from Tables 1 and 2. Figure 5 displays the log CR and 1/T linear regression plots. The preexponential factor k, and the computed activation energies E^*^_a_,. The adjustment of the corrosion process's mechanism in the presence of adsorbed inhibitor molecules may be used to explain changes in the apparent activation energies' values [37]. The literature [38–40] extensively discusses the fluctuation of the apparent activation energy E^*^_a_ in the presence and absence of MOFs. MOFs were discovered to either cause E^*^_a_ values to rise or fall. In the presence of the MOFs, the apparent activation energy was higher than it was in the absence of the inhibitor [41]. In our investigation, E^*^_a_ rises as MOFs concentration rises, and all values of E^*^_a_ were higher than they would have been without MOFs. At normal temperatures, this sort of inhibitor effectively delays corrosion; but, at higher temperatures, the inhibitor's effectiveness is reduced. According to Arrhenius law (Eq. 2), corrosion rate will rise with temperature. E^*^_a_ may change as well. According to the results, A and E^*^_a_ seem to rise steadily as the inhibitor's concentration rises”. Using an alternative formulation of Arrhenius equation [42] it is possible to access additional kinetic information (enthalpy and entropy of corrosion process):

6 $${\text{CR}}\, = \,{\text{RT}}/{\text{ N}}_{{\text{A}}} {\text{h exp }}\Delta {\text{S}}^{*} /{\text{ R exp}} - \Delta {\text{H}}^{*} /{\text{RT}}$$

**Fig. 5:**
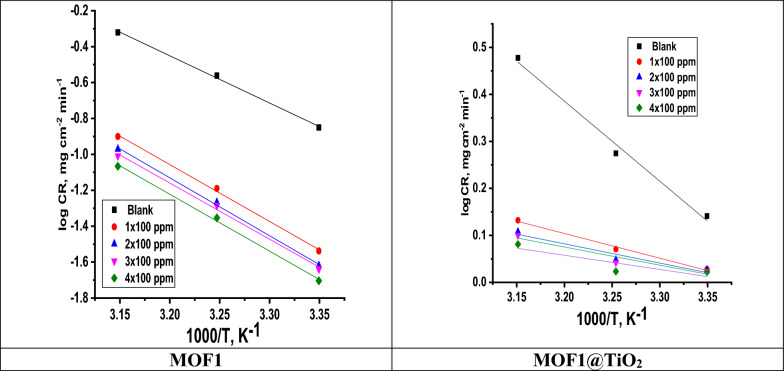
Arrhenius plots of the CR for both the 1 M HCl (Blank) and the various concentrations solution of MOF1 and MOF@TiO_2_

where N_A_ stands for Avogadro's number, h is Planck's constant, and ∆H^*^ and ∆S^*^ stand for the enthalpy and entropy of activation, respectively. “A plot of ln (CR/T) vs 1/T can be seen in Fig. 6. Straight lines are produced, from which the values of ∆H^*^ and ∆S^*^ are determined and listed in Table 3 with a slope of (-∆H^*^/R) and an intercept of (ln(R/Nh) + (∆S^*^/R)). The process of dissolving Al is endothermic, as indicated by the + ve sign of ∆H^*^. According to a study of Table 3's data, the values of E^*^_a_ and ∆H^*^ rise with inhibitor concentration, indicating that the presence of MOFs raises the energy barrier for corrosion reactions. This indicates that the corrosion reaction will continue to be driven toward surface locations that exhibit steadily increasing E^*^_a_ values in the presence of the inhibitor. The entropy of activation ΔS in the absence and presence of the inhibitor is large and negative”. This suggests that the activated complex in the rate-determining phase reflects an association rather than a dissociation step, i.e., when one moves from reactants to the activated complex, there is a reduction in disordering [43].

**Fig. 6:**
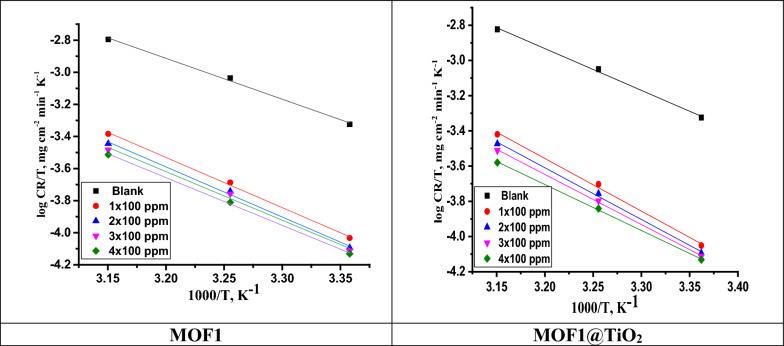
Variation of log (corrosion rate/T) versus 1000/T for the dissolution of Al in the absence and presence of various doses of MOF1 and MOF@TiO_2_

**Table 3:** Arrhenius and transition-state parameters for the dissolution of Al in both 1 M HCl (Blank) and the various doses solution of MOF1 and MOF@TiO_2_

Compound	Conc. ppm	E_a_^*^ kJ mol^−1^	-∆H^*^ kJ mol^−1^	-∆S^*^ J mol^−1^ K^−1^
Blank with Al	0.0	40.7	38.1	97.0
MOF1 With CS	100	52.6	49.5	73.3
	200	54.8	52.2	74.7
	300	55.2	52.9	81.1
	400	56.4	53.8	85.2
MOF1@TiO_2_	100	55.9	53.4	82.5
	200	57.4	54.8	86.6
	300	59.4	56.6	87.0
	400	62.9	60.2	90.6

* Activation

#### Adsorption isotherm

A study of the adsorption isotherm is necessary to investigate how inhibitors inhibit metals by adsorbing on their surfaces. “It is often useful to use the Frumkin, Temkin, Freundlich, and Langmuir isotherms to determine the best corrosion inhibitor adsorption rates [44, 45]. The best-fitting isotherm for the experimental data was determined using the correlation coefficient (R^2^). The MOFs' adsorption on the Al surface is thought to have followed this isotherm based on the strong correlation (R^2^ > 0.99) between the two variables”. The linear form of Langmuir isotherm is represented in Eq. (7) [46].

7 $$\frac{{\text{C}}}{{\uptheta }} = { }\frac{1}{{{\text{K}}_{{{\text{ads}}}} }} + {\text{c}}$$

**Fig. 7:**
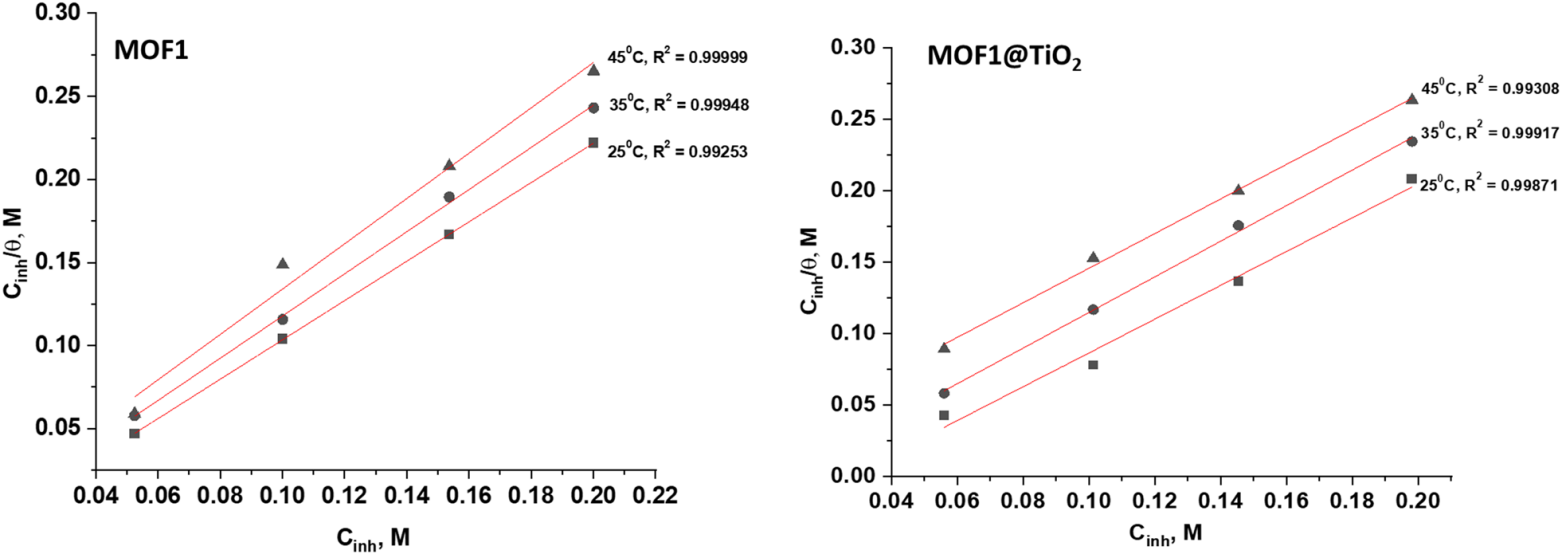
“Langmuir adsorption isotherms for the MOFs in 1.0 M HCl solution at 25 ^°^C”

**Fig. 8:**
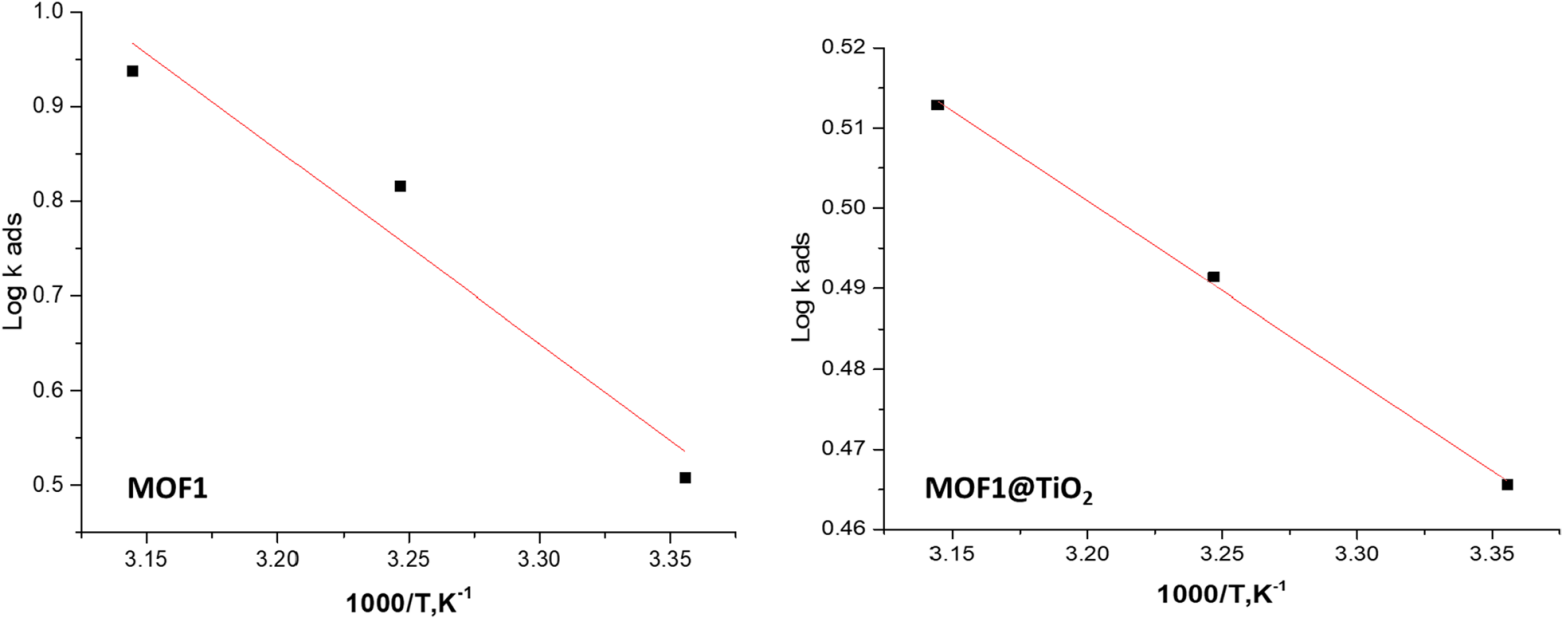
Enthalpy plot of MOFs inhibitors for Al in 1.0 M HCl solution

Figure 7 illustrates the Langmuir plot with an intercept, and using ${{\text{K}}}_{{\text{ads}}}$= 1/intercept, a ${{\text{K}}}_{{\text{ads}}}$ value was calculated for MOF1 and MOF1@TiO_2_. This was used in Eq. 7 to calculate Gibbs free energy of adsorption ($\Delta {{\text{Go}}}_{{\text{ads}}}$). Table 4 shows the obtained adsorption parameters and Gibbs free energy of adsorption [47].

8 $$\Delta {\text{Go}}_{{{\text{ads}}}} = { } - {\text{RTln}}\left( {55.5{\text{ x K}}_{{{\text{ads}}}} } \right)$$

where, “R = 8.314 J mol^−1^ K^−1^is the experimental temperature while 55.5 is concentration of H_2_O in mol. L^−1^. According to this isotherm, there are no interactions between the adsorbed species and they each occupy a single site. There is a general trend that corrosion inhibitors with high ${{\text{K}}}_{{\text{ads}}}$ values and small $\Delta {{\text{Go}}}_{{\text{ads}}}$ values offer better corrosion protection. According to Table 4, the order of the ${{\text{K}}}_{{\text{ads}}}$ and $\Delta {{\text{Go}}}_{{\text{ads}}}$ values of MOFs are MOF1@TiO_2_ > MOF1, which indicates that η (MOF1) < η (MOF1@TiO_2_), the same as the experimental results. The physical adsorption is due to the electrostatic attraction between the corrosion inhibitor and metal at $\Delta {{\text{Go}}}_{{\text{ads}}}$ values lesser or equal to − 20 kJ mol^−1^. Chemisorption takes place when $\Delta {{\text{Go}}}_{{\text{ads}}}$ values are more than or equal to − 40 kJ mol^−1^ and occur when electrons are transferred between metal and corrosion inhibitor molecule. Adsorption types consisting of physical and chemical adsorption occur when $\Delta {{\text{G}}}_{{\text{ads}}}$ is between − 20 and − 40 kJ mol^−1^ [48]. In our studies according the obtained results MOF1@TiO_2_ behave as mixed adsorption (chemical and physical) but MOF1 behave as mainly physical adsorption one. Additionally, the negative values of ∆G^o^_ads_ demonstrate the strong binding of the inhibitor molecule onto the surface of Al [49]. The negative values of ∆G^o^_ads_ indicate that the adsorption of MOFs is spontaneous process and the stability of the adsorbed layer on the Al surface. A straight line was drawn (Fig. 8) on the graph with a slope of -ΔH^o^/2.303R [50]. An enthalpy value of 41.9 kJ mol^−1^ is produced by physisorption, while approximately 100 kJ mol^−1^ is produced by chemisorption” [51, 52]. While MOF1 exhibits physisorption with a ∆H^o^ value less than 100 kJ mol^−1^, MOF1@TiO2 exhibits mixed type (chemisorption and physisorption) with a ∆H^o^ > 100 kJ mol^−1^.

**Table 4:** Thermodynamic coefficients of adsorption of MOFs on Al substrate in 1.0 M HCl

Compd	Temp., °C	K_ads_, M^−1^	Log K_ads_	− ΔG^o^_ads_ kJ mol^−1^	−ΔH^o^_ads_ kJ mol^−1^	ΔS^o^_ads_ J mol^−1^K^−1^
MOF1	25	− 1.15066	0.0609	10.80	88.0	34.6
	35	− 1.16331	0.0657	10.67		34.7
	45	− 1.45069	0.1615	11.60		36.5
MOF1@TiO_2_	25	− 1315.1	3.1189	29.75	134.0	92.7
	35	− 1996.57	3.3002	27.75		96.6
	45	− 34.4708	3.5374	32.98		62.8

### PDP study

**Fig. 9:**
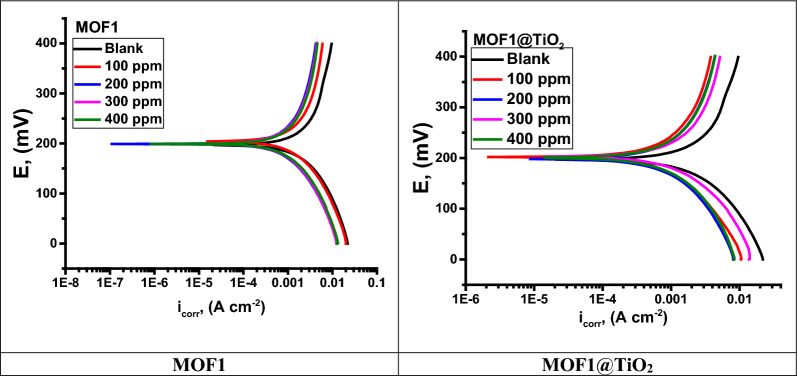
Plots of PDP curves for Al dissolution with and without MOFs

The kinetics of cathodic and anodic reactions were investigated by polarization experiments. A Tafel plot was used to extrapolate the corrosion current densities (${{\text{i}}}_{{\text{corr}}}$) of inhibitors at different concentrations (100, 200, 300, and 400 ppm) by extrapolation of cathodic (β_c_) and anodic (β_a_) branches of Tafel plots. “Fig. 9 shows the polarization profile of Al in 1.0 M HCl. An MOF inhibitor shifts both anodic and cathodic curves towards lower current densities. Table 5 shows that, as inhibitors were added, ${{\text{i}}}_{{\text{corr}}}$ values decreased because surface coverage increased [53]. Surfaces of the Al adsorbed inhibitor functional groups, increasing their effectiveness as inhibitors. Moreover, inhibitors inhibited hydrogen evolution as well as metal dissolution. When the inhibitors are added to the Al, the selected ${{\text{E}}}_{{\text{corr}}}$ values adjust towards more + ve values, indicating that MOFs inhibit corrosion of Al at 25 ^°^C. The tested inhibitor is classified as anodic or cathodic when the change in ${{\text{E}}}_{{\text{corr}}}$ is greater than 85 mV [31]. The highest displacement of MOFs is 385 mV, suggesting that adding an acidic.

**Table 5:** Corrosion parameters for Al corrosion in one molar HCl with and without MOFs concentrations

Comp	Conc. ppm	i_corr._ µA/ cm^2^	E_corr._, mV/SCE	β_a_ mV/dec	-β_c_ mV/dec	C.R mpy	θ	η%
Blank	0.0	5.714	566.86	2981	136.8	186.58	–	–
MOF1	100	4.965	737.36	586.8	134.8	162.12	0.131	13.1
	200	2.719	731.39	224.5	99.0	61.164	0.524	52.4
	300	1.873	738.62	128.1	75.4	43.973	0.672	67.2
	400	1.347	740.45	309.4	117.4	88.789	0.764	76.4
MOF1@TiO_2_	100	1.936	533.03	84.0	58.2	25.522	0.694	69.4
	200	1.793	601.50	204.7	109.1	58.564	0.716	71.6
	300	1.631	737.38	176.1	79.3	63.219	0.742	74.2
	400	0.750	735.07	161.8	99.1	53.248	0.876	86.9

### EIS study

**Fig. 10:**
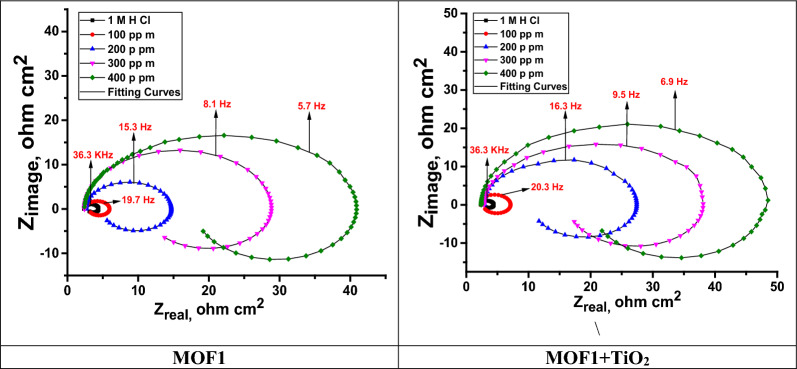
The Nyquist plot illustrates the corrosion behavior of Al in one molar HCl at 298 K, both in the presence and absence of MOFs inhibitors

**Fig. 11:**
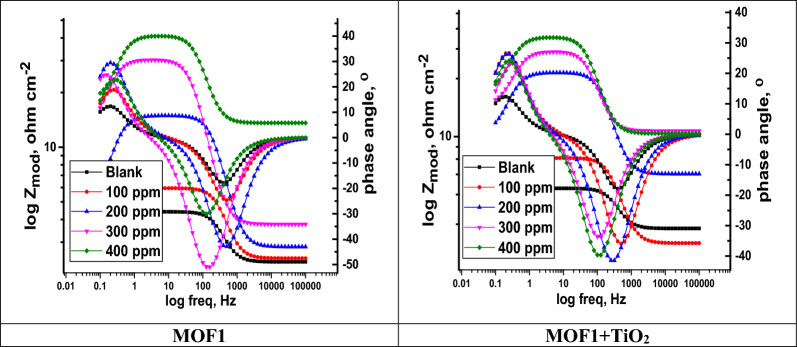
Bode curves for Al alloy in the lack and existence of diverse doses of MOFs inhibitors at 25 °C

**Fig. 12:**
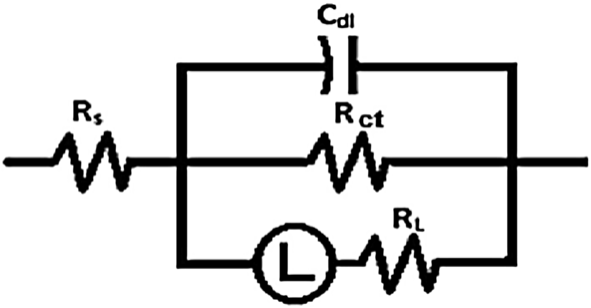
Electrical comparable circuit employed to fit the impedance information

An EIS measurement was carried out to assess how the surface of the Al acted in the studied solution. The EIS method was used to analyses inhibitors at different concentrations (100, 200, 300, and 400 ppm). “EIS experimental data was calculated using Gamry Analyst software, including charge transfer resistance (R_ct_), and double-layer charge (${{\text{C}}}_{{\text{dl}}}$). The significant results are reported in Table 6. It was found that ${{\text{R}}}_{{\text{ct}}}$ values increased as corrosion inhibitor concentrations increased, which indicates the corrosion inhibitor molecules form a protective layer on Al samples, while high charge transfer resistance corresponds to systems that corrode slowly. Figure 10 shows a Nyquist plot of the impedance spectra, showing the diameter of semi-circular rises with rising of MOFs concentration indicating the adsorption of MOFs on Al surface and creating protective films. Figure 11 demonstrates the Bode plots for Al in 1 M HCl in the lack and existence of diverse doses of MOFs. It was found that as the MOFs concentration rises the total impedance Z increases as well as the phase angle shifts to more value due to the adsorption of MOFs on Al surface. The circuit equivalent model appears in Fig. 12 which was utilized to show the attained impedance information. This demonstration includes the solution resistance (R_s_), the charge-transfer resistance of the interfacial corrosion reaction (R_ct_), the inductance (L), the inductive resistance (R_L_), and the double layer capacitance (C_dl_)”. A good fit with this demonstration was achieved by outcomes information. When an inductive circle appears the resistance of polarization can be computed from the subsequent Eq. (6) [54]:

9 $$R_{P} = \frac{{R_{ct} \times R_{L} }}{{R_{ct} + R_{L} }}$$

**Table 6:** An EIS parameter of Al in 1.0 M HCl with MOF inhibitors at room temperature

Comp	Conc., × 10^4^ M	R_ct_, Ω cm^2^	C_dl_, µFcm^−2^	R_L_ Ω cm^2^	R_p_, Ω cm^2^	L Hcm^−2^	θ	$\mathrm{\%IE}$	Goodness of Fit $\chi$ ^2^
Blank	–	4.99	57.35	3.07	1.90	8.31	–	–	5.62 × 10^–3^
MOF1	100	9.55	48.84	3.14	2.36	9.67	0.477	47.7	5.44 × 10^–3^
	200	15.12	42.11	4.31	3.35	10.33	0.670	67.0	5.33 × 10^–3^
	300	30.25	39.45	11.5	8.33	22.59	0.835	83.5	4.81 × 10^–3^
	400	38.92	35.19	14.8	10.72	33.93	0.872	87.2	3.17 × 10^–3^
MOF1@TiO_2_	100	14.11	44.08	4.85	3.61	11.45	0.646	64.6	3.48 × 10^–3^
	200	27.42	38.41	10.15	7.41	19.13	0.818	81.8	4.84 × 10^–3^
	300	38.15	34.15	13.52	9.98	26.72	0.869	86.9	5.15 × 10^–3^
	400	46.89	31.20	21.02	14.51	34.11	0.894	89.4	5.52 × 10^–3^

EIS data indicate that the R_p_ magnitudes increment and the C_dl_ magnitudes diminish with the increment of the MOFs doses, Table 6. “This is often owing to the progressive substitution of H_2_O atoms by the adsorption of MOFs atoms on the surface of the metal and diminishing the degree of the disintegration response. The large R_P_ magnitudes are for the most part related to a slower corrosion system. The high-frequency capacitive circuit was caused by charge transfer reactions and surface inhomogeneity, while the low-frequency inductive circuit could be caused by the adsorption of H_ads_^+^, Cl^−^ molecules and corrosion inhibitors on the Al surface”. The double-layer capacitance is calculated using Eq. [55];

10 $${\text{C}}_{{{\text{dl}}}} = { }\frac{1}{{2{\pi f}_{{{\text{max}}}} {\text{R}}_{{{\text{ct}}}} }}$$

The values of ${{\text{C}}}_{{\text{dl}}}$ values gradually decrease with “MOF presence compared to the values of the blank sample. When inhibitors adsorb on the Al surface and/or the local dielectric constant decreases, the electrical double-layer thickness increases, resulting in a decrease in ${{\text{C}}}_{{\text{dl}}}$ values” [56]. The corrosion inhibition performance parameters obtained from mass loss, EIS, and polarization studies are almost similar.

### Surface analysis

#### Scanning electron microscope (SEM) analysis

**Fig. 13:**
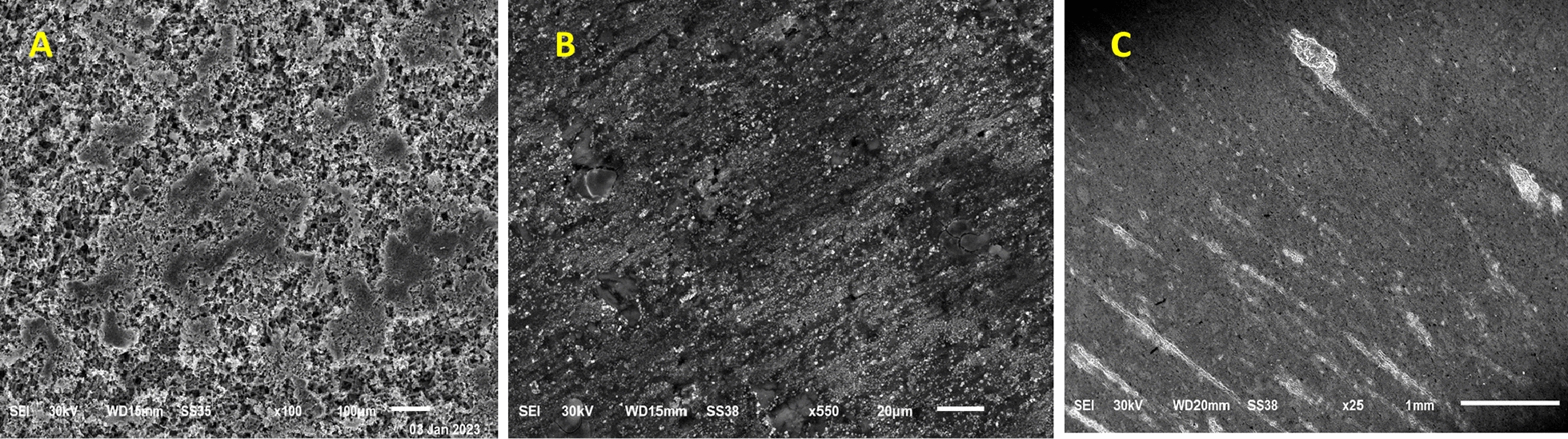
The SEM micrographs show the surface of Al after being immersed in different solutions for 24 h. In image **A**, Al was immersed in 1.0 M HCl. According to image **B**, Al was immersed in 1.0 M HCl with 400 ppm MOF1. As seen in image **C**, Al was immersed in 1.0 M HCl at 25 °C and 400 ppm MOF1@TiO_2_

Figure 13 shows the results of immersing Al samples in a solution containing 1.0 M HCl for 24 h. “The experiment was conducted with and without the presence of inhibitors, namely MOF1 and MOF1@TiO2. In Fig. 13A, the SEM micrograph shows a rough surface of AL in HCl without an inhibitor, indicating corrosion of the AL [57]. Figure 13B–C reveal that the surface coverage of the AL samples increases with the addition of 400 ppm concentrations of MOF1@TiO_2_ and MOF1 inhibitors”. This implies that a protective layer is created on the metal surface, inhibiting the corrosion of Al [58].

#### Energy dispersive X-ray analysis (EDX)

**Fig. 14:**
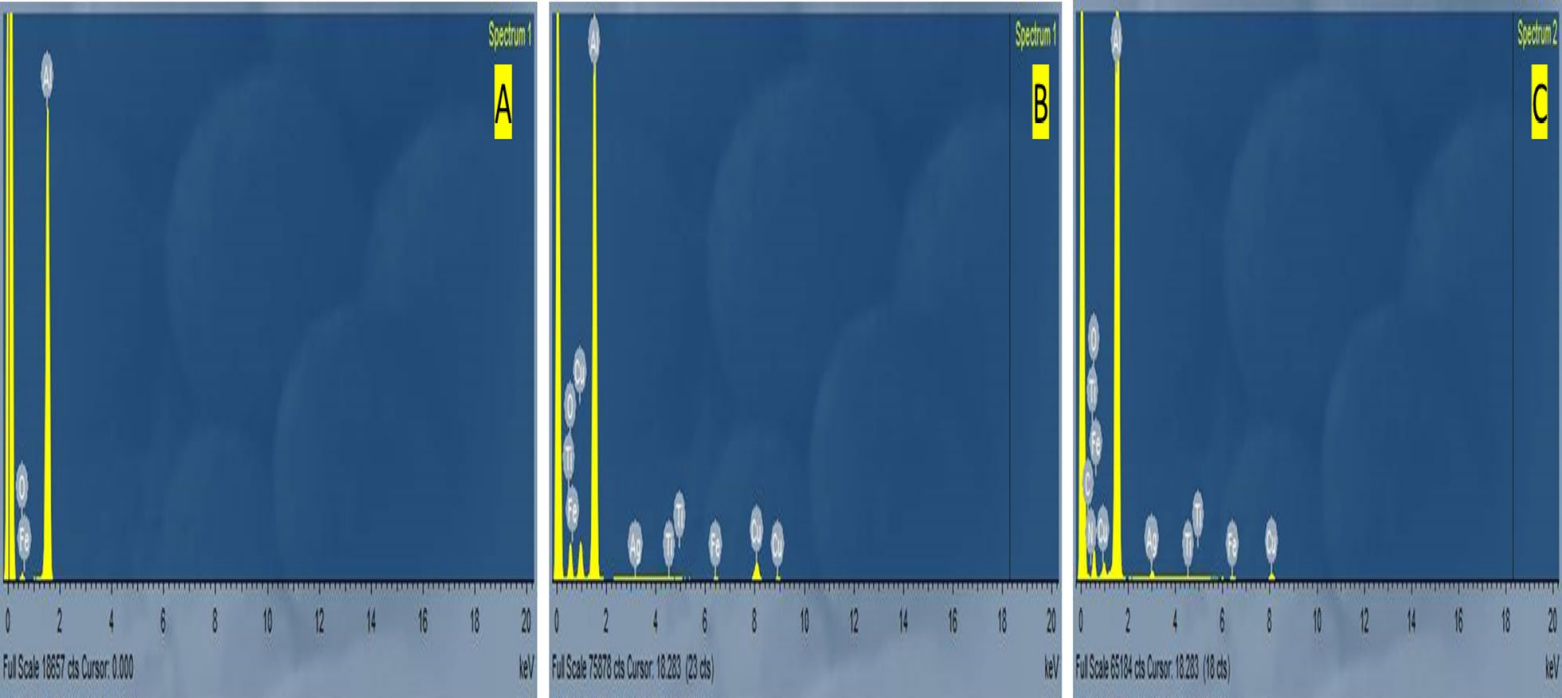
EDX spectra of Al in 1.0 M HCl (Blank) and 400 ppm MOF1 and MOF1@TiO2

Al samples were subjected to an energy dispersive X-ray (EDX) examination under two conditions: a six-hour immersion in a 1.0 M hydrochloric acid (HCl) solution (1) without Cu-MOFs and (2) with an optimal concentration of Cu-MOFs (400 ppm). The results have been obtained from the EDX spectra are shown in Fig. 14. Without any inhibitors, the EDX spectra of Al reveal the primary constituent of the elements already present on the surface of Al” (Al, Fe, O). However, additional peaks of (Ti) atoms may be seen, which serve as the active centers of these inhibitors for adsorption and film formation on the Al surface (C,N,Fe,Ti,O, Ag, Cu).

#### AFM analysis

**Fig. 15:**
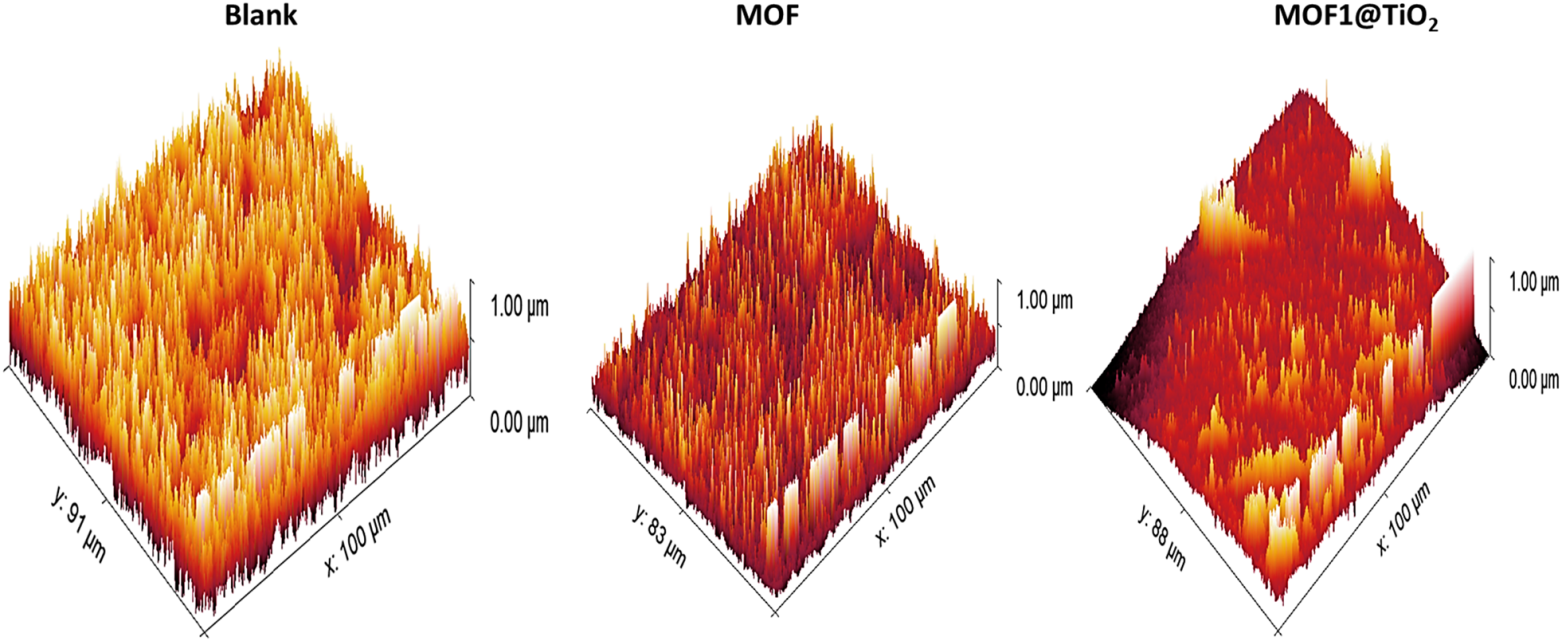
AFM analyses on CS with and absence of 21 × 10^–6^ M SCPs for 1 day’s involvement

One may assess the surface roughness of coupons on an angstrom scale thanks to the atomic or near-atomic resolution surface topography afforded by the AFM technique. This approach is effective for examining the surface analysis of both inhibited and uninhibited Al surfaces. A 3-D AFM image of the exterior of Al subjected to 1.0 M HCl for 24 h with and without the optimal MOFs level measured is shown in Fig. 15. After being submerged in 1.0 M HCl without an inhibitor for 24 h, the common roughness of the surface of Al was measured and determined to be 284.37 nm. In Fig. 15, a 3D image of the Al surface is shown while being exposed to the highest doses (400 ppm) of MOF1 and MOF1@TiO_2_, bringing the roughness to 125.7 and 121.2 nm, respectively”. The outcomes approach demonstrates the accumulation of MOFs on the Al surface and the application of a protective coating.

#### Quantum chemical parameters

**Fig. 16:**
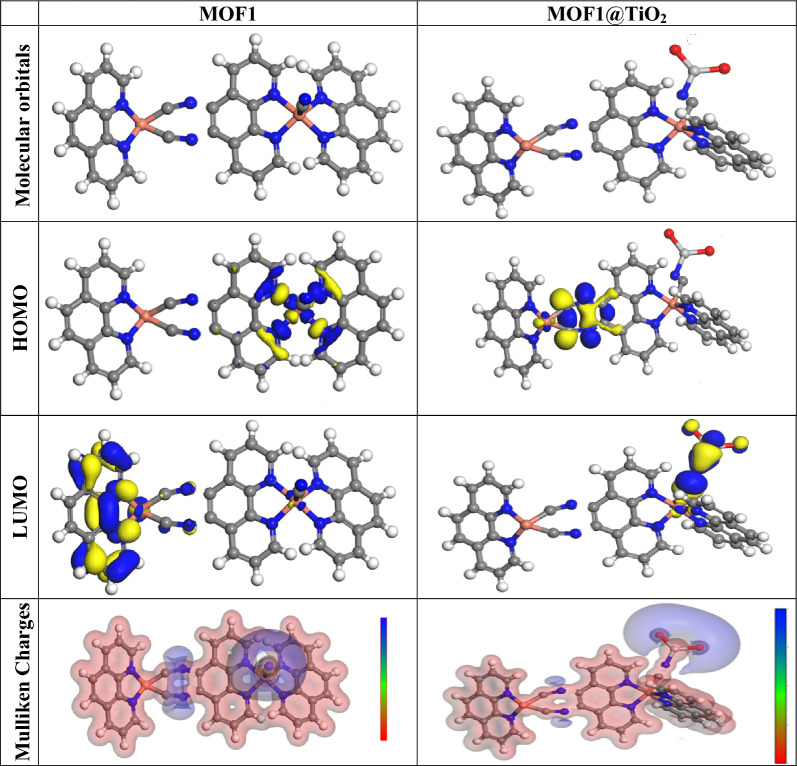
The optimized geometrical structure, (HOMO), and (LUMO) of the tested MOFs at DMol3

The lower energy band gap value, which is represented in the energy band gap ΔE_g_ (ΔE = E_HOMO_ E_LUMO_), “indicates that organic molecules are highly reactive and exhibit excellent corrosion behaviour on the surface of Al. An analysis of the impact of MOFs molecule's orientation on inhibition performance was conducted using density function theory (DFT). As shown in Fig. 16, the optimized geometry, HOMO surface, and LUMO surface of studied inhibitors can be found. The parameters HOMO (E_H_), LUMO (E_L_), and dipole moment (μ) for MOFs gradients were directly obtained from DFT. Equations (11)–(16) were used to calculate the energy gap (ΔE), electronegativity (χ), global hardness (η), global softness (σ), the fraction of electron transfer (ΔN) and back-donation (ΔE back-donation)”, was calculated as Koopmans’s theorem [59] from the next balance (Table 7):

11 $$\mu = - \chi = - \frac{{I_{p } + E_{A} }}{2}$$

12 $$\chi = \frac{{I_{p } + E_{A} }}{2}$$

13 $$\eta = \frac{{I_{P - } E_{A} }}{2}$$

14 $$\sigma = \frac{1}{\eta }$$

15 $$\omega = \frac{{\mu^{2} }}{2\eta }$$

16 $$\Delta E_{back\, donation} = - \,\frac{\eta }{4}$$

**Table 7:** Quantum chemical data for MOFs under study

Compound	MOF1	MOF1@TiO_2_
*-E*_*HOMO*_*, eV*	3.981	3.622
*-E*_*LUMO*_*, eV*	3.656	3.458
*ΔE, eV*	0.325	0.164
*I*_*P*_*, eV*	3.981	3.622
*E*_*A*_*, eV*	3.656	3.458
$\chi$*, eV*	3.8185	3.54
*η, eV*	0.1625	0.082
*σ, eV*	6.153846	12.19512
*ω*	44.86444	76.4122
*∆N*	9.789231	21.09756
*ΔE*_*back-donation*_	− 0.04063	− 0.0205
*Dipole moment (Debye)*	27.35	23.27

Numerous articles have discussed how higher values of “E_HOMO_ and lower values of E_LUMO_ determine the greater electron-donating and accepting abilities of an inhibitor. Inhibitors are more reactive when a lesser value of ΔE is present. In this instance, MOF1@TiO_2_ ΔE value is lower while higher values for MOF1. In comparison to MOF molecules, these values suggest that MOF1@TiO_2_ molecule has a high degree of reactivity. Metals and inhibitors can be understood using the number/fraction of electron transfer (ΔN). If the ΔN value of an inhibitor is higher, it is found to have a stronger capability of donating electrons to metallic surfaces. Compared to MOFs molecules, MOF1@TiO_2_ exhibits greater amounts of ΔN in the gaseous phase, indicating that MOF1@TiO_2_ exhibits a stronger inhibitory effect”.

#### Monte Carlo (MC) simulation

**Fig. 17:**
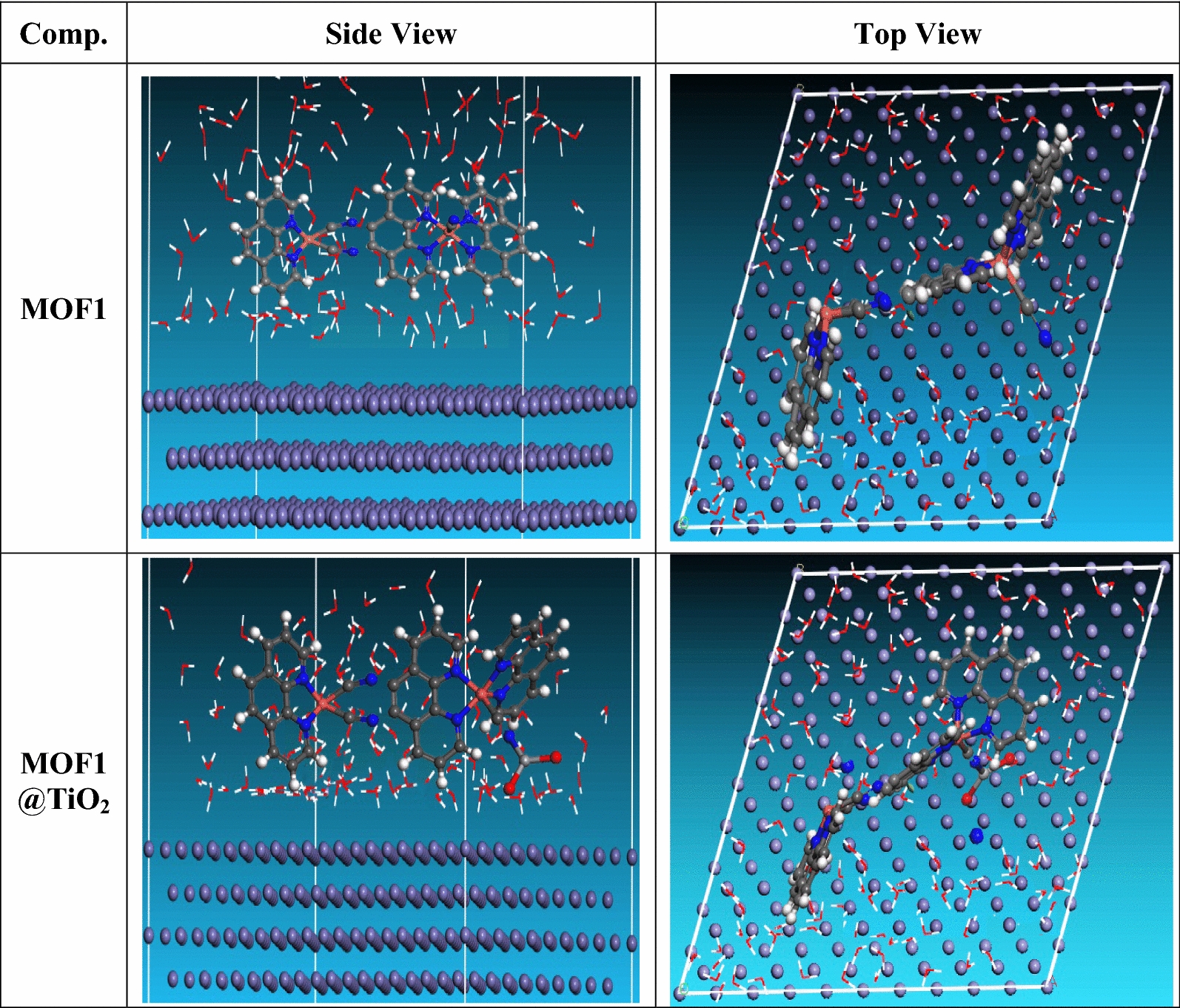
Adsorption configurations of the MOFs molecules on Al surface

Monte Carlo simulation was utilized to find out more about the interactions between the molecules under study and the metal surface in an acidic environment. “Views of the more sturdy arrangement for the adsorption of MOFs on the surface of cleaved Al (110) from the top and sides (Fig. 17). MC stimulation done by adsorption lactor module detect the interaction between inhibitors and surface area of Al (1 1 0) crystal with discovering the best adsorption sites [60]. Choosing the Al (110) plane was based on its best stability and well-packed structure. Forcite module was used to optimize the geometry of MOFs. The Simulation annealing was used to calculate fine-quality adsorption using five cycles of 50,000 steps. This study investigates low-energy configurations of Al (110)-inhibitor system in aqueous solution. In order to simulate corrosion in a real-life scenario, the simulation was conducted in an aqueous environment with water molecules. Table 8 presents the adsorption configuration of the nearly parallel in position resulting from relaxation of the inhibitor molecule on Al (110). The descriptors computed from MC stimulation are in Table 8. The tabulated adsorption energies are − 4129.697 and − 4090.575 kcal/mole for MOF1@TiO_2_, MOF1 respectively. The outputs show that the two inhibitors are efficient adsorptive inhibitors taking in respect that the better one is MOF1@TiO_2_ which is attuned with the experimental results”. Based on theoretical modeling it’s obvious that MOFs based proved to be powerful inhibitors for the Al which is confirmed by experimental and spectral investigation.

**Table 8:** Monte Carlo simulation parameters of adsorption of MOFs molecules on Al (110) surface

Structures	Adsorption energy	Rigid adsorption energy	Deformation energy	Compound dE_ad_/dNi	H_2_O dE_ad_/dNi
Al (1 1 0)/Inhibitor MOF1/H_2_O	− 4090.575	− 4268.145	177.57	− 232.81	− 12.73
Al (1 1 0)/Inhibitor MOF1@TiO_2_/H_2_O	− 4129.697	− 4312.247	182.55	− 274.47	− 14.31

### Mechanism of corrosion inhibition

The adsorption process is influenced by various factors, including the interaction between Al and the corrosive solution, the electrochemical potential, the chemical composition, and the surface properties of Al [61]. “An adsorbent typically sticks to a surface by forming bonds or through chemical or physical adsorption [62]. When the conditions are acidic, heteroatoms like nitrogen, sulfur, and oxygen become protonated [63]. This was confirmed by MOF1 which was physically adsorbed on Al surface due to this might get adsorbed onto Al surface by van der Waals force to form a protective film to isolate Al from corrosion. The number of electrons surrounding the active center of a compound determines its inhibitory activity”[64]. As for MOF1@TiO_2_ Nano-structure inhibitor, two possible mechanisms are demonstrated. First, when N and O heteroatoms are protonated, they attach themselves to the negatively charged surface of Al, which was reabsorbed Cl^−^ ions [65]. Additionally, the second mechanism is the neutral species may adsorb on metal surface via the chemisorption mechanism, involving the displacement of water molecules from the metal surface and the sharing electrons between the N and O atoms and Al. In the majority of cases, the percentage of MOF1@TiO_2_ is higher than that of MOF1. This is due to its higher molecular size, which may cover more areas. Also, due to the presence of TiO_2_ which expected that due to the ionic sites in its surface as a result of occupying MOF1 surface by (TiO_2_).

## Conclusions

The findings showed that in a 1 M HCl solution, MOF1 and its modified surface with TiO_2_, known as MOF@TiO2, inhibited Al corrosion. By raising the two inhibitors' concentrations, the inhibition effectiveness was raised and reduced by raising temperature. The inclusion of MOF1 and MOF1@TiO2 did not alter the mechanism of the hydrogen evolution process or the Al dissolution, according to potentiodynamic polarization curves. The inhibitors function as inhibitors of mixed types. The adsorption process was physically, and this finding was corroborated by the Henry isotherm data for these chemicals. Additionally, the inhibitory effect of the two inhibitors on Al is confirmed by SEM, EDX, and AFM studies. The PDP, EIS, and ML test findings validate that MOF1@TiO_2_ is a more potent corrosion inhibitor than MOF1 and are generally in excellent accord with surface investigation. Additionally, greater adsorption on the metal surface caused by the presence of TiO_2_ on the Al surface improved the corrosion inhibitor's action. The experimental findings and the theoretical ones agreed rather well. This finding implies that the studied MOFs are a good option for inhibiting Al corrosion in HCl solution.

## Data Availability

All data generated or analyzed during this study are included in this published article.
